# Epidemiology and clinical outcomes of common respiratory viruses detected by multiplex RT-PCR in patients at a national infectious disease reference center in Romania, 2022–2025

**DOI:** 10.1186/s12985-026-03217-y

**Published:** 2026-06-29

**Authors:** Ovidiu Vlaicu, Dragoș Florea, Leontina Banică, Simona Paraschiv, Oana Săndulescu, Anca Cristina Drăgănescu, Anca Streinu-Cercel, Adrian Streinu-Cercel, Dan Oțelea, Victor Daniel Miron

**Affiliations:** 1 Microbiology Department, Faculty of Biology, University of Bucharest, Bucharest, Romania; 2 National Institute for Infectious Disease “Prof. Dr. Matei Balș”, Bucharest, Romania; 3 Carol Davila University of Medicine and Pharmacy, Bucharest, Romania; 4 Society for Research and Innovation in Infectious Diseases, Bucharest, Romania

**Keywords:** ARIs, respiratory viruses, Romania, RT-PCR multiplex, surveillance

## Abstract

**Introduction:**

A broad range of common respiratory viruses contributes substantially to the burden of acute respiratory infections (ARIs) across all age groups. However, data on their epidemiology, seasonality, and clinical impact in Eastern Europe remain limited.

**Methods:**

We conducted a retrospective laboratory-based surveillance study including patients evaluated for ARIs at the National Institute for Infectious Diseases “Prof. Dr. Matei Balș”, Bucharest, Romania, between November 2022 and October 2025. Respiratory samples were tested using multiplex real-time RT-PCR panels detecting multiple respiratory viruses. The present analysis focused on adenovirus (AdV), enterovirus (HEV), rhinovirus (HRV), parainfluenza virus (PIV), human coronaviruses (CoV), human bocavirus (HBoV), and human metapneumovirus (MPV). Epidemiological characteristics, detection patterns, and clinical outcomes were analyzed.

**Results:**

Among 4930 patients tested, 1709 (34.7%) had at least one of the studied viruses detected, corresponding to 2144 viral detections (22.2% patients with viral codetections). HRV was the most frequently detected virus. Also, viral codetections most commonly involved HRV. Analysis of monthly detections across the three seasons studied demonstrated virus-specific temporal patterns. Specifically, MPV demonstrated a more consistent winter–spring detection pattern, whereas HRV was detected throughout the study period. Overall, 50.2% of patients required hospitalization, with the highest rates observed among patients with CoVs and MPV detection. Requirement of supplemental oxygen was more common in patients hospitalized with MPV (28.4%) and PIV (24.6%). Patients with viral codetections had higher hospitalization rates compared with those with monodetections (57.7% vs. 48.1%; OR = 1.47; *p* < 0.001), although no significant differences were observed in requirement of supplemental oxygen, ICU admission, or length of hospital stay.

**Conclusions:**

AdV, HEV, MPV, PIV, HBoV, CoV and HRV accounted for a substantial proportion of viral detections among patients evaluated for ARIs in routine clinical practice. The predominance of HRV and the frequent occurrence of viral codetections highlight the complexity of respiratory viral epidemiology revealed by multiplex molecular diagnostics. Broader surveillance of respiratory viruses may improve understanding of respiratory infection dynamics in the post-pandemic period.

## Introduction

Acute respiratory infections (ARIs) remain major global public health concerns and represent substantial causes of morbidity and mortality worldwide. In Europe alone, ARIs are estimated to account for more than 145,000 deaths annually [1].

For certain respiratory viruses, such as SARS-CoV-2 and influenza viruses, effective preventive strategies, including vaccination are available, and are followed by reduction in disease severity and mortality when administered early [2]. In contrast, for most other respiratory viruses, no targeted antiviral therapies or prevention intervention are currently available. Clinical management relies primarily on supportive treatment, based on symptom control and monitoring for complications. Because of this therapeutic gap, respiratory viruses continue to contribute substantially to healthcare utilization and hospital admissions.

The COVID-19 pandemic profoundly altered the epidemiology of respiratory viruses worldwide [3–5]. Public health measures, including lockdowns and social distancing, disrupted established seasonal patterns and led to atypical circulation dynamics. Prior to the pandemic, seasonal human coronaviruses (CoVs) and human metapneumovirus (MPV) typically peaked during winter months, whereas human adenovirus (AdV), human rhinovirus (HRV), human enterovirus (HEV), and human parainfluenza virus (PIV) circulated throughout the year [3, 6]. Pandemic-related mitigation strategies particularly impacted the transmission of enveloped viruses such as PIV and MPV, resulting in shifts in seasonal distribution and fluctuating incidence patterns [7]. Understanding the post-pandemic behavior of these viruses is essential for anticipating healthcare burden and optimizing surveillance strategies.

A broad range of common respiratory viruses represent major contributors to pediatric respiratory disease, accounting for approximately 60–70% of cases in children, with HRV, AdV, MPV, and PIV among the most frequently identified pathogens [8–10]. However, the clinical relevance of co-detections remains incompletely understood, particularly in the context of evolving respiratory viral circulation patterns.

The clinical presentation of respiratory viral infections is often nonspecific. Due to largely overlapping symptoms, differentiation between viral etiologies cannot be done solely based on clinical presentation. Multiplex real-time reverse transcription polymerase chain reaction (RT-PCR) assays enable simultaneous detection of multiple respiratory pathogens and are essential for accurate etiological diagnosis, especially in hospitalized patients and high-risk populations [10]. While rapid diagnostic assays for SARS-CoV-2, influenza, and RSV are widely available in emergency settings, comprehensive testing for other respiratory viruses remains less routinely implemented, potentially delaying accurate etiological diagnostics and appropriate clinical management.

In our institution, previous studies have already described the epidemiology of influenza viruses, RSV, and SARS-CoV-2 [11–15]. Therefore, the present study focuses specifically on other respiratory viruses detected by multiplex RT-PCR. The aim of this study was to characterize the epidemiology and clinical outcomes associated with a broad range of common respiratory viruses among patients evaluated for ARIs in a tertiary infectious disease hospital in Romania during three consecutive seasons (2022–2025).

## Methods

### Study design and setting

We performed a retrospective, laboratory-based surveillance study of viral respiratory tract infections at the National Institute for Infectious Diseases “Prof. Dr. Matei Balș”, Bucharest, Romania. This is the largest infectious disease hospital in Romania and a national reference center for diagnosis, management, and research on infectious diseases. The study period covered 1 November 2022 through 31 October 2025. Surveillance was based on clinician-ordered multiplex RT-PCR testing performed as part of routine clinical care, without additional sampling or testing procedures implemented for research purposes.

### Study population and inclusion criteria

All respiratory specimens collected from patients with ARI evaluated at the National Institute for Infectious Diseases “Prof. Dr. Matei Balș” during the study period were eligible for inclusion if they were tested using a multiplex RT-PCR respiratory panels. Both inpatients and outpatients, across all age groups, were included in the laboratory-based analysis.

For the purposes of the present analysis, we report data on the following range of respiratory viruses: AdV, HEV, MPV, PIV, HBoV, CoV, HRV. For patients from whom multiple respiratory samples were collected during the same hospitalization episode, only the first specimen was included, to avoid duplicate counting. Clinical data were analyzed separately for hospitalized patients, for whom more detailed clinical information was available from hospital records.

### Specimen types and laboratory testing

Respiratory specimens were obtained according to routine clinical practice and consisted of upper respiratory tract samples, including nasopharyngeal or nasal swabs. All samples were collected using viral transport medium (VTM) (Copan Italia S.P.A., Brescia, Italy) and processed at the National Institute for Infectious Diseases “Prof. Dr. Matei Balș” Molecular Diagnostics Laboratory. Viral RNA was extracted from 280µL VTM using MagMAX™ Viral/Pathogen Nucleic Acid Isolation Kit (ThermoFisher Scientific) and KingFisher Sample Purification System (ThermoFisher Scientific). Detection of respiratory viruses was performed by RT-PCR using the Allplex™ Respiratory Panel 1 (influenza A virus, influenza A-H1, influenza A-H1pdm09, influenza A-H3, influenza B virus, respiratory syncytial virus A, respiratory syncytial virus B), Panel 2 (human adenovirus - AdV, human enterovirus - EV, human metapneumovirus - MPV, human parainfluenza viruses - PIV 1, 2, 3, 4), Panel 3 (human bocavirus - HBoV, human coronaviruses - CoV 229E, NL63, OC43, human rhinovirus - HRV) assays (Seegene, Seoul, South Korea), in accordance with the manufacturer’s instructions.

### Data collection and definitions

Clinical and demographic data were retrospectively extracted from medical records using a standardized data collection form. Variables collected included age, sex and clinical setting (inpatient or outpatient). For hospitalized patients’ additional data were collected, including length of hospital stay, occurrence of respiratory failure with requirement for supplemental oxygen, and admission to the intensive care unit (ICU).

Patients were grouped into three seasons, corresponding to national-level monitoring: November 2022-October 2023 (2022/23 season), November 2023-October 2024 (2023/24 season), and November 2024-October 2025 (2024/25 season). Age was categorized into three groups: children (0–17 years), adults (18–64 years), and elderly (≥ 65 years). In particular, children were divided into five age subgroups: infants (< 1 year), toddlers (1–2 years), preschoolers (3–4 years), school children (5–13 years) and teenagers (14–17 years).

### Statistical analysis

Statistical analysis was performed using IBM SPSS Statistics for Windows, version 25 (IBM Corp., Armonk, NY, USA). Descriptive data were presented as frequency (n, N) and percentage (%). The chi-squared test (χ^2^) with risk calculation by odds ratio (OR) and 95% confidence interval (95%CI) was used to analyze categorical variables. For continuous variables, we presented mean with standard deviation (SD) or median with interquartile range (IQR) according to their distribution. The level of statistical significance was set at *p* < 0.05.

## Results

### Epidemiological data

**Fig. 1:**
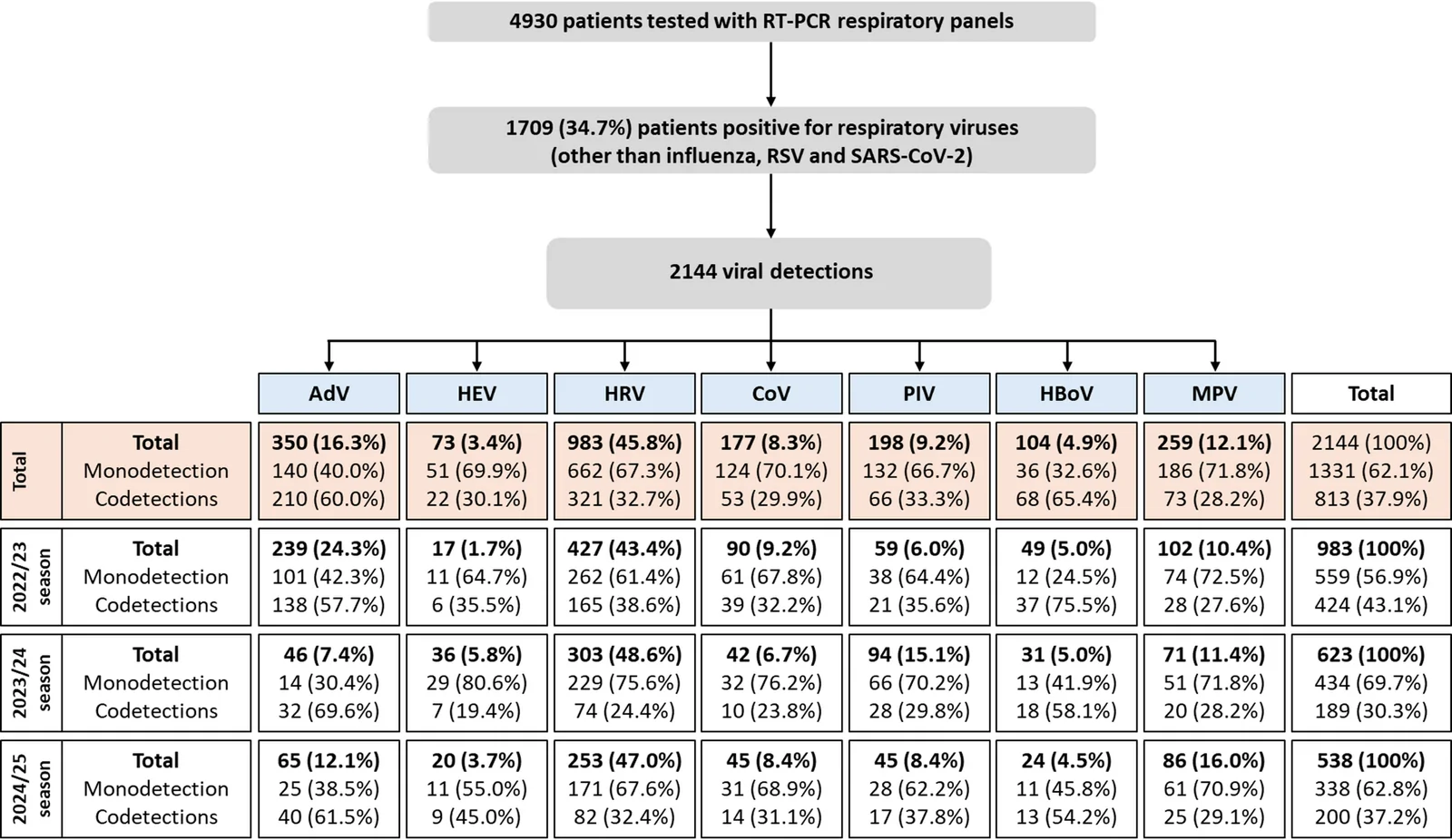
Distribution of viral detections identified by multiplex RT-PCR among patients with acute respiratory infections and their distribution across the three study seasons (2022–2025). Percentages of each virus types were calculated relative to the total positive detections. AdV - human adenovirus, HEV - human enterovirus, HRV - human rhinovirus, CoV - common coronaviruses, PIV - parainfluenza virus, HBoV - human bocavirus, MPV - human metapneumovirus

During the study period, a total of 4930 patients were tested using multiplex RT-PCR panels for respiratory viruses. Among these, 1709 (34.7%) were positive for at least one of the following viruses: AdV, HEV, HRV, PIV, CoVs, HBoV, MPV. Overall, 2144 viral detections were identified among these patients, corresponding to 1331 patients with monodetections (77.8%) and 378 patients with codetections (22.2%) (329 double, 41 triples, and 8 quadruple detections). HRV had the highest detection rate in all three seasons analyzed (43.4%, 48.6%, and 47.0%, respectively). AdV and HBoV were more frequently identified in codetections than in monodetections, regardless of season (60.0% and 65.4%, respectively). The overall distribution of virus-specific detections is presented in Fig. 1.

**Fig. 2:**
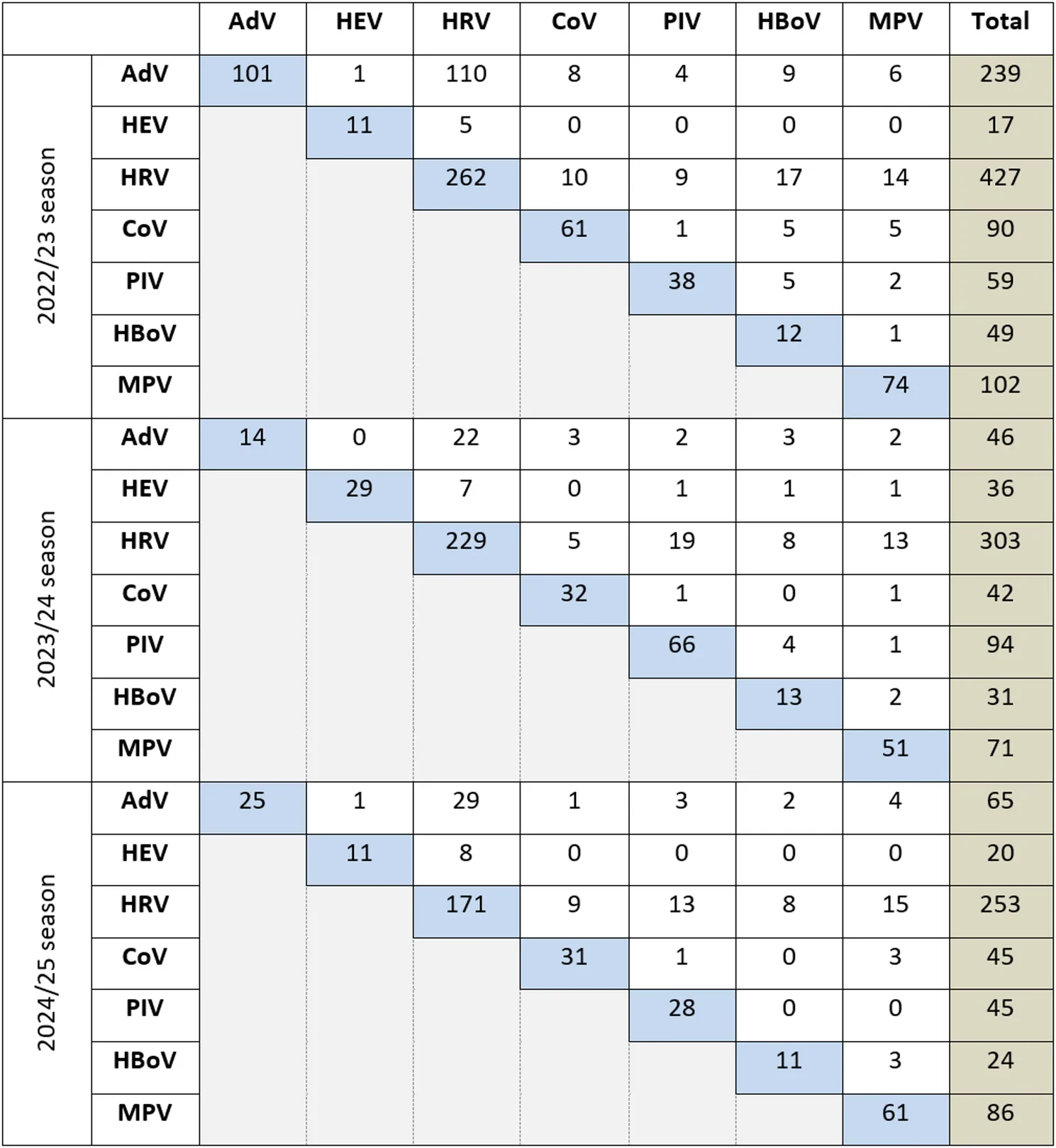
Frequency of viral mono- and co-detections detected among patients with acute respiratory infections by season. AdV - human adenovirus, HEV - human enterovirus, HRV - human rhinovirus, CoV - common coronaviruses, PIV - parainfluenza virus, HBoV - human bocavirus, MPV - human metapneumovirus

Across the three seasons, codetections were consistently dominated by combinations involving HRV. The most frequently observed codetections included HRV with AdV. Codetections involving CoVs and MPV, or HEV were less common and were also typically observed in association with HRV. Overall, the pattern of viral codetection was comparable across the three seasons, with variations mainly reflecting changes in the overall number of detected cases (Fig. 2).

**Fig. 3:**
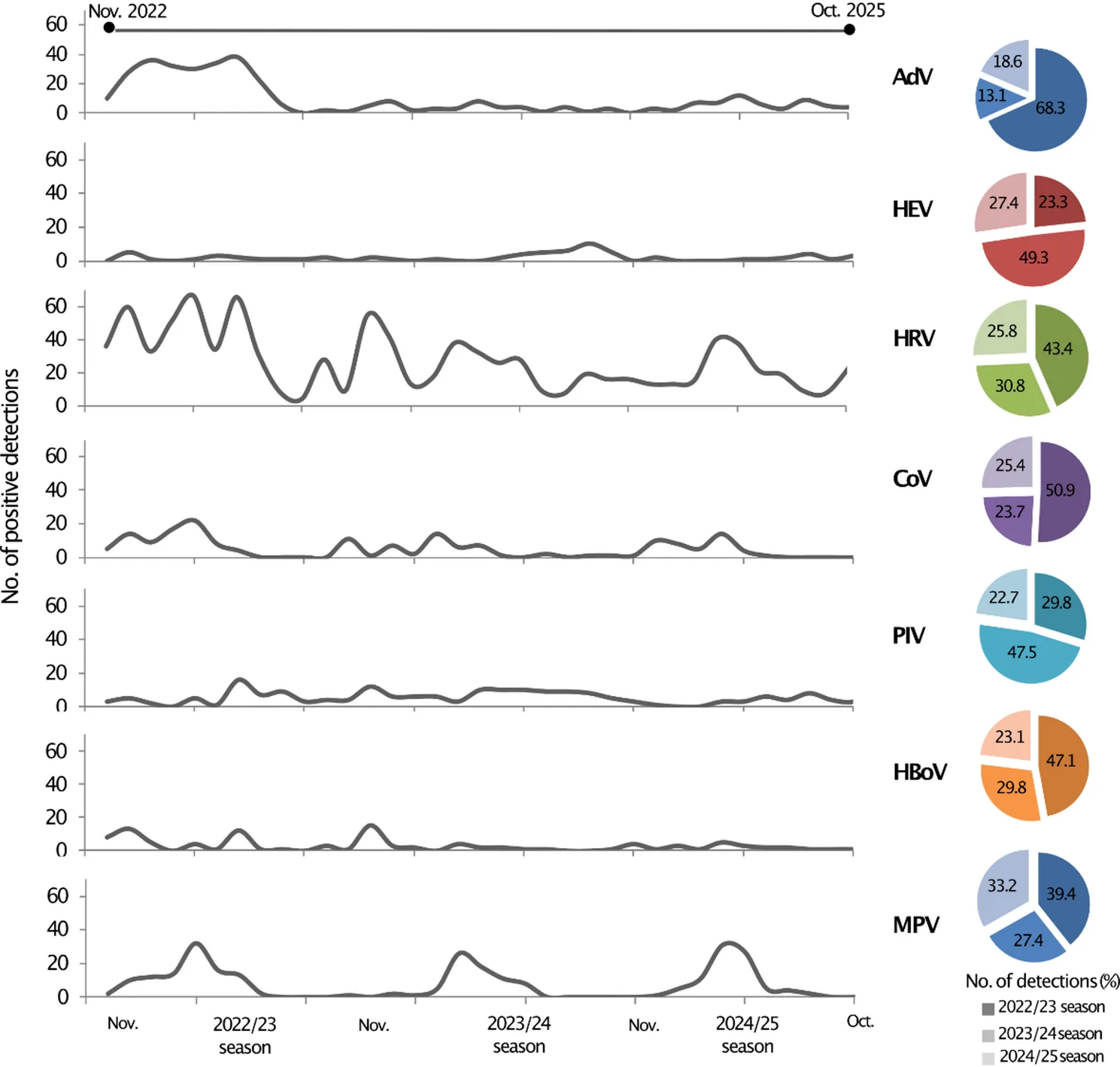
Temporal distribution of viral detections during the study period (November 2022 - October 2025). AdV - human adenovirus, HEV - human enterovirus, HRV - human rhinovirus, CoV - common coronaviruses, PIV - parainfluenza virus, HBoV - human bocavirus, MPV - human metapneumovirus

Analysis of monthly detections over the three seasons demonstrated virus-specific temporal patterns. The temporal distribution of detection for studied respiratory viruses is illustrated in Fig. 3. HRV was detected throughout the entire observation period and accounted for the largest proportion of detections in all seasons, with multiple peaks occurring each year. AdV detections were most frequent during the 2022/23 season, with a marked early-season peak followed by substantially lower detection counts in subsequent seasons. CoVs and PIV showed variable monthly detection without a consistent seasonal peak. HBoV detections occurred sporadically and remained relatively infrequent throughout the study period. In contrast, MPV exhibited a more defined temporal pattern, with peaks typically observed during the winter-spring months.

**Fig. 4:**
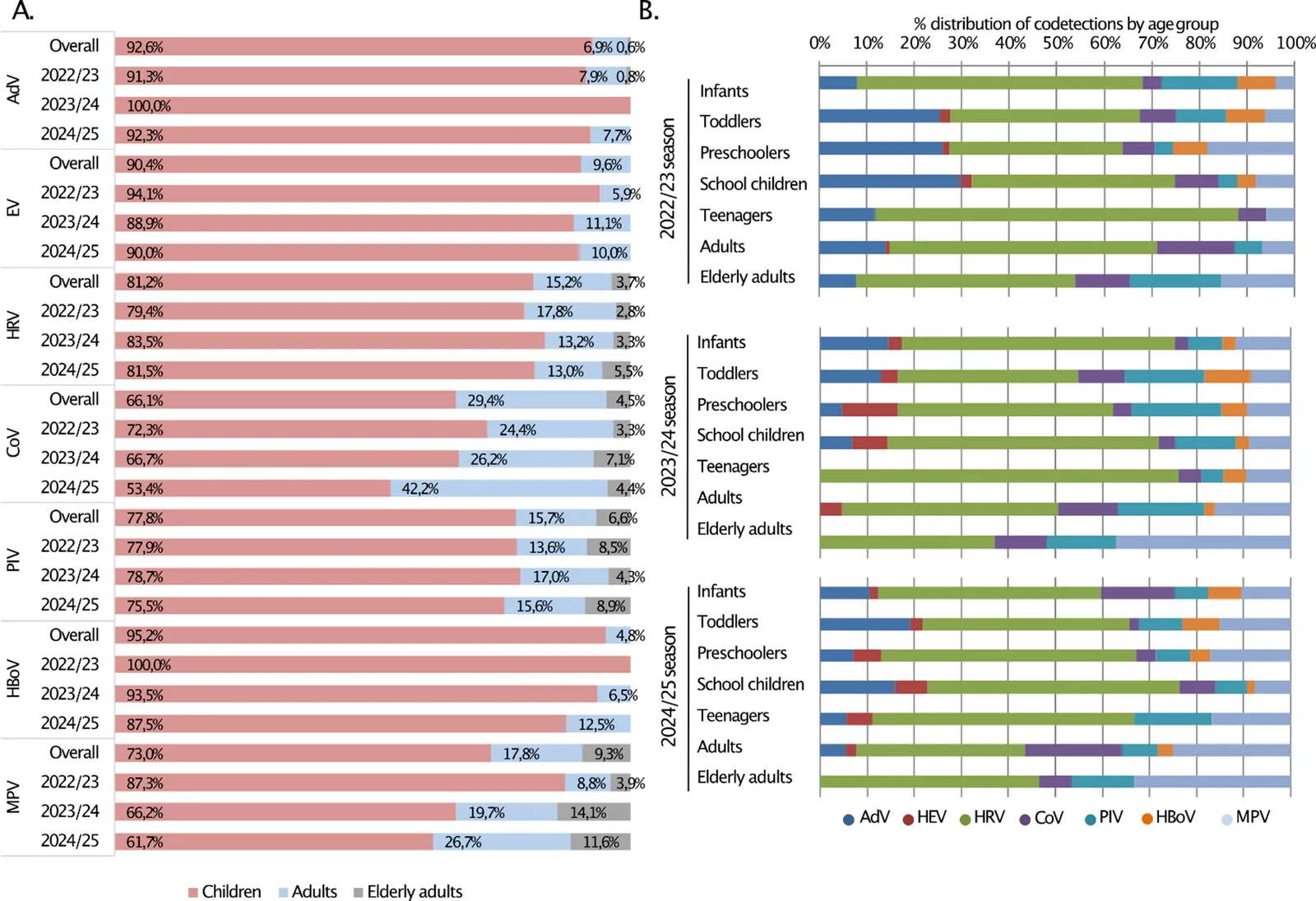
Age distribution of viral detections among patients with acute respiratory infections.** (A) **Proportion of viral detections by age group. **(B) **Distribution of viral monodetections across pediatric age subgroups and study seasons. AdV - human adenovirus, HEV - human enterovirus, HRV - human rhinovirus, CoV - commoncoronaviruses, PIV - parainfluenza virus, HBoV - human bocavirus, MPV - human metapneumovirus

Most viral detections were from pediatric patients (*n* = 1747/2144, 81.5%), while detections in elderly adults only represented 3.9% (*n* = 83/2144). The majority of respiratory viruses showed a strong detection in pediatric population, with more than 90% of HBoV (95.2%), AdV (92.6%) and HEV (90.4%) detections occurring in children. In contrast, CoVs (29.4% in adults and 4.5% in elderly adults) and MPV (17.8% in adults and 9.3% in elderly adults) were detected across a wider age range, including adults and elderly adults (Fig. 4A). The relative contribution of each virus within age groups and season is presented in Fig. 4B. Although the absolute number of detections varied between seasons, the age-specific distribution patterns for each virus remained largely consistent over time.

### Clinical data

The hospitalization rate was 50.2% for the entire group of patients analyzed, with 16.1% requirement of supplemental oxygen and 2.0% ICU admission rate (Table 1). Patients with viral codetections had significantly higher hospitalization rates compared to those with monodetections (57.7% vs. 48.1%; OR = 1.47; 95%CI:1.17–1.85; *p* = 0.001). However, codetections were not associated with increased rates of requirement of supplemental oxygen (17.0% vs. 15.8%; OR = 1.32; 95%CI:0.89–1.96; *p* = 0.166) or ICU admission (2.8% vs. 1.9%, OR = 1.47, 95%CI: 0.52–4.21; *p* = 0.466). Among hospitalized patients with viral monodetections, rates differed significantly by viral etiology (*p* < 0.001, χ^2^(6) = 48.66, V = 0.078), with the highest rates observed MPV (62.4%) and CoVs (61.3%), and the lowest for EV (23.5%). Also, rates of requirement of supplemental oxygen differed significantly between patients according to etiology (*p* < 0.001, χ^2^(6) = 30.1, V = 0.061), whereas ICU admission did not vary significantly (*p* = 0.443). Among patients with viral codetections, no statistically significant differences were observed in hospitalization, requirement of supplemental oxygen, or ICU admission rates according to the number of detected viruses (all *p* > 0.05).

**Table 1:** Clinical outcome according to type of virus and codetections

Type of virus	Total number	Inpatients *N* (%)^a^	Requirement of supplemental oxygen *N* (%) ^b^	ICU admission *N* (%)^b^	Days of hospitalization median (IQR)
**Monodetections**	1331	640 (48.1)	101 (15.8)	12 (1.9)	4 (2, 6)
AdV	140	74 (52.6)	6 (8.1)	2 (2.7)	4 (3, 5.5)
HEV	51	12 (23.5)	2 (16.7)	0 (0.0)	3.5 (2, 4)
HRV	662	277 (41.8)	38 (13.7)	5 (1.8)	4 (2, 5.5)
CoV	124	76 (61.3)	3 (3.9)	0 (0.0)	3.5 (2.5, 5)
PIV	132	65 (49.2)	16 (24.6)	0 (0.0)	3 (4, 6.5)
HBoV	36	20 (55.6)	3 (15.0)	1 (5.0)	5 (4, 6)
MPV	186	116 (62.4)	33 (28.4)	4 (3.4)	5 (2, 7)
**Codetections**	378	218 (57.7)	37 (17.0)	5 (2.8)	4 (3, 6)
2 viruses	329	185 (56.2)	29 (15.7)	5 (2.7)	5 (3, 6.5)
3 viruses	41	27 (65.9)	6 (22.2)	1 (3.7)	4 (3, 6)
4 viruses	8	6 (75.0)	2 (33.3)	0 (0.0)	4.25 (± 1.3)^c^
**Overall**	1709	858 (50.2)	138 (16.1)	17 (2.0)	4 (3, 6)

^a^- The denominator is the total number for each virus; ^b^ - The denominator is the total number of inpatients for each virus; ^c^- Mean and standard deviation (SD). AdV - human adenovirus, HEV - human enterovirus, HRV - human rhinovirus, CoV - common coronaviruses, PIV - parainfluenza virus, HBoV - human bocavirus, MPV - human metapneumovirus

The median length of hospitalization was comparable between patients with mono- and co-detections, with a median of 4 days in both groups (monodetections: 4 days [IQR: 2–6 days]; codetections: 4 days [IQR:3–6 days], *p* > 0.05). No clinically meaningful differences in length of stay were observed according to viral etiology or number of detected viruses (all *p* > 0.05).

## Discussion

In this laboratory-based surveillance study conducted over three consecutive seasons (2022–2025), we describe the epidemiological patterns and clinical outcome of seven common respiratory viruses identified in patients from the largest infectious diseases centre from Bucharest, Romania. Our findings contribute to the limited data available from Eastern Europe regarding the epidemiology of respiratory viruses. Because the present study is based on clinician-ordered multiplex RT-PCR testing, the observed patterns should be interpreted as detection patterns within the tested population rather than direct estimates of viral circulation at the population level.

Respiratory viruses (AdV, HEV, MPV, PIV, HBoV, CoV, HRV) accounted for over one-third (*n* = 1709, 34.7%) of infections in positive patients highlighting their substantial contribution to respiratory morbidity. Nearly half of all viral detections occurred during the 2022/23 season, indicating a post-pandemic rebound in respiratory viral circulation. Seasonal analysis demonstrated virus-specific temporal dynamics, with distinct annual predominance patterns among them. Although respiratory viral circulation largely followed a cold-season pattern, all viruses showed summer activity as well.

HRV was the predominant pathogen across all seasons, accounting for nearly half of all viral detections. This observation is consistent with numerous pre- and post-pandemic studies identifying HRV as the most frequently detected respiratory virus in both outpatient and hospital-based cohorts [7, 8, 16, 17]. HRV is characterized by year-round circulation and rapid resurgence following relaxation of COVID-19 mitigation measures, particularly due to its non-enveloped structure and environmental stability [8, 17]. Despite its high incidence, HRV was not associated with the highest rates of hospitalization or requirement of supplemental oxygen in our cohort, supporting previous data suggesting that HRV contributes substantially to case numbers but less consistently to severe disease burden [8, 18].

In contrast, MPV and CoV were associated with the highest hospitalization rates among patients with viral monodetections. Our data align with existing literature indicating that MPV is an important cause of lower respiratory tract infection not only in children but also in adults and elderly individuals [19–21]. In our study, MPV also demonstrated relatively consistent temporal patterns across the three seasons, with peaks occurring during the winter-spring period. Some previous reports describe a seasonal predominance of MPV infections in late winter and early spring [20, 22]. Similarly, common coronaviruses such as OC43 and NL63 have been reported to cause clinically significant disease in vulnerable populations, particularly older adults with comorbidities [23, 24]. In our cohort, although CoVs detections frequently associated hospitalization, the rates of requirement of supplemental oxygen and ICU admission remained relatively low, suggesting that hospitalization may have been influenced by age, comorbidities, or clinical precaution rather than true severe respiratory compromise.

Viral codetections were identified in more than one-third of positive patients, a proportion comparable to that reported in pediatric and multiplex PCR-based studies [25]. Patients with codetections had higher odds of hospitalization compared to those with monodetection (OR = 1.47). However, codetections were not associated with significantly increased rates of requirement of supplemental oxygen or ICU admission. This pattern suggests that while codetection may influence the decision to hospitalize, it does not necessarily translate into greater clinical severity. Similar findings have been reported in systematic reviews indicating that viral codetections are common but not consistently associated with worse clinical outcomes [26, 27]. Importantly, many codetections in our cohort involved HRV, which has previously been described to exhibit prolonged viral shedding and frequent codetection, raising the possibility that some codetections may not reflect simultaneous active pathogenic processes [28]. Overall, in multiplex RT-PCR-based studies, the detection of multiple viruses may reflect prolonged viral shedding or incidental detection rather than simultaneous active infection, which complicates the interpretation of viral codetections [29, 30].

Notably, the number of detected viruses (double versus triple or quadruple infections) was not significantly associated with differences in hospitalization, requirement of supplemental oxygen, or ICU admission. Although a numerical increase in severity indicators was observed with increasing viral count, this trend did not reach statistical significance and likely reflects the limited number of multiple-virus cases. Previous studies have similarly shown inconsistent associations between the number of codetected viruses and disease severity, particularly after adjustment for age and comorbidities [27].

Age distribution patterns in our study were stable across seasons. Most detections occurred in pediatric patients, particularly for HBoV, AdV, and EV, consistent with established epidemiology [8, 31, 32]. In contrast, MPV and CoV showed broader age distribution, including adults and elderly individuals, supporting their role as clinically relevant pathogens beyond childhood [19, 24, 33]. The predominance of viral detections in children likely reflects both the well-established higher burden of respiratory viral infections in pediatric populations and the testing practices within our institution. Multiplex RT-PCR testing is more frequently requested for children with acute respiratory infections, particularly in hospitalized cases, which may contribute to higher proportions of pediatric detections, as observed in our dataset [8, 34].

Length of hospital stay was similar across patients with mono- and co-detections and did not differ meaningfully by viral etiology. This finding further supports the notion that host-related factors may contribute more substantially to the clinical course than viral etiology alone [8, 34, 35].

The present study has several limitations that should be acknowledged when interpreting the findings. First, its retrospective and single-center design may limit generalizability, as the study population reflects patients evaluated in a tertiary infectious diseases hospital, where admission thresholds and testing practices may differ from those in primary or secondary care settings. Second, multiplex RT-PCR detects viral nucleic acid but does not distinguish between active infection and prolonged viral shedding, which may be particularly relevant for viruses such as HRV and HBoV. Consequently, some coinfections may represent codetection rather than true simultaneous pathogenic interaction. In addition, the use of clinician-ordered multiplex testing may introduce selection bias, as patients undergoing multiplex PCR are more likely to represent more severe or diagnostically uncertain cases. Consequently, the detection frequencies observed in this study should not be interpreted as population-level incidence estimates.

The main strengths of this study include the multi-season design, the standardized molecular diagnostics performed in a single reference laboratory, the inclusion of a broad age range, and the integrated analysis of epidemiological patterns, codetection profiles, and clinical outcomes.

Taken together, our findings highlight that a broad range of respiratory viruses account for a substantial proportion of viral detections among patients evaluated for acute respiratory infections in routine clinical practice. The consistent predominance of rhinovirus and the frequent occurrence of viral codetections illustrate the complex viral landscape revealed by multiplex molecular diagnostics, particularly in pediatric populations.

## Conclusions

This study provides a comprehensive description of the epidemiology, detection patterns, and clinical outcomes associated with AdV, HEV, HRV, PIV, CoVs, HBoV, MPV among patients evaluated for acute respiratory infections in a specialized infectious disease hospital in Romania. Our findings highlight the substantial contribution of these viruses to respiratory infections detected in clinical practice, with HRV representing the most frequently identified pathogen across all seasons. Viral codetections were common, but not associated with increased clinical severity, suggesting that multiple molecular detections should be interpreted cautiously in the clinical context. These results emphasize the importance of including a broader spectrum of respiratory viruses in diagnostic and surveillance strategies. Future multicenter and population-based studies integrating virological, clinical, and epidemiological data will be essential to better define the true burden and clinical impact of these pathogens in the post-pandemic era. 

## Data Availability

The datasets generated and analyzed during the current study are available from the corresponding author upon reasonable request.
